# Acute systemic DNA damage in youth does not impair immune defense with aging

**DOI:** 10.1111/acel.12478

**Published:** 2016-04-13

**Authors:** Jason L. Pugh, Sarah A. Foster, Alona S. Sukhina, Janka Petravic, Jennifer L. Uhrlaub, Jose Padilla‐Torres, Tomonori Hayashi, Kei Nakachi, Megan J. Smithey, Janko Nikolich‐Žugich

**Affiliations:** ^1^ Department of ImmunobiologyUniversity of Arizona College of MedicineTucsonAZUSA; ^2^ Arizona Center on AgingUniversity of Arizona College of MedicineTucsonAZUSA; ^3^ Graduate Interdisciplinary Program in GeneticsUniversity of ArizonaTucsonAZUSA; ^4^ Centre for Vascular ResearchUniversity of New South WalesSydneyNSW 2052Australia; ^5^ Radiation Effects Research FoundationMinato‐KuHiroshimaJapan; ^6^ The BIO5 InstituteUniversity of ArizonaTucsonAZUSA; ^7^ Present address: Departments of Structural Biology and Microbiology & ImmunologyStanford University School of Medicine291 Campus DriveStanford, CA, 94305USA; ^8^ Present address: Translational Neurotrauma Research ProgramBarrow Neurological Institute at Phoenix Children's HospitalDepartment of Child HealthUniversity of Arizona College of Medicine550 E Van Buren StPhoenixAZ 85004USA

**Keywords:** aging, DNA damage, irradiation, T‐cell, vaccination

## Abstract

Aging‐related decline in immunity is believed to be the main driver behind decreased vaccine efficacy and reduced resistance to infections in older adults. Unrepaired DNA damage is known to precipitate cellular senescence, which was hypothesized to be the underlying cause of certain age‐related phenotypes. Consistent with this, some hallmarks of immune aging were more prevalent in individuals exposed to whole‐body irradiation (WBI), which leaves no anatomical repository of undamaged hematopoietic cells. To decisively test whether and to what extent WBI in youth will leave a mark on the immune system as it ages, we exposed young male C57BL/6 mice to sublethal WBI (0.5–4 Gy), mimicking human survivor exposure during nuclear catastrophe. We followed lymphocyte homeostasis thorough the lifespan, response to vaccination, and ability to resist lethal viral challenge in the old age. None of the irradiated groups showed significant differences compared with mock‐irradiated (0 Gy) animals for the parameters measured. Even the mice that received the highest dose of sublethal WBI in youth (4 Gy) exhibited equilibrated lymphocyte homeostasis, robust T‐ and B‐cell responses to live attenuated West Nile virus (WNV) vaccine and full survival following vaccination upon lethal WNV challenge. Therefore, a single dose of nonlethal WBI in youth, resulting in widespread DNA damage and repopulation stress in hematopoietic cells, leaves no significant trace of increased immune aging in a lethal vaccine challenge model.

**Abbreviations**
Gy: gray (unit of radiation)
RWN: repliVAX west nile
T_N_: naïve T cells
WBI: whole‐body irradiation
WNV: west nile virus

## Introduction

Infectious disease remain one of the top ten leading causes of death in people over 65 (NCHS, 2013). The elderly are particularly susceptible to emergent or ‘never‐before‐seen’ pathogens, such as the West Nile virus (WNV) (Murray *et al*., 2006), severe acute respiratory syndrome (Leung *et al*., 2004) and chikungunya virus (Borgherini *et al*., 2007). Vaccine efficacy is also dramatically decreased in the elderly (McElhaney, 2002). Despite the presence of other factors, including, but not limited to, changes in microbial colonization with aging, the reduced function of barrier tissues, and hormonal, proteostatic, metabolic, and other changes with aging, it is believed that a common and variable decline in immunity, called immune aging or immune senescence, decisively contributes to these changes. Both innate and adaptive immune systems are affected by aging (rev. in Haynes & Swain, 2012; Kogut *et al*., 2012; Nikolich‐Zugich, 2014). Age‐associated T‐cell defects include decreased naïve T‐cell (T_N_) abundance and proportional increase in memory cells, reduction in proliferation and function (rev. in Maue *et al*., 2009; Haynes & Swain, 2012; Nikolich‐Zugich, 2014), and inability to defend against intracellular infections (Brien *et al*., 2009; Smithey *et al*., 2011; Jiang *et al*., 2013). Pathogen‐specific antibody production and efficacy also decrease with age (Frasca & Blomberg, 2009; Kogut *et al*., 2012).

Irradiation‐induced damage to genetic material has been shown to cause cell cycle arrest and repair responses for over fifty years (Painter & Robertson, 1959; Hollaender & Curtis, 1975). However, some of the arrested cells did not die, nor repair to re‐enter cell cycle. Rather, they survived for prolonged periods of time and, similar to the phenotype described by Hayflick on fibroblast doublings in culture, were deemed to be ‘senescent’ (Hayflick & Moorhead, 1961). From there, a subset of theories of biological aging suggested that many cells, including immune cells, may have defective functions in old age because of a lifetime of self‐renewal and DNA damage‐related senescence (Hasty *et al*., 2003; Campisi, 2005; d'Adda di Fagagna, 2008; Lopez‐Otin *et al*., 2013). However, the role of DNA damage in biological aging (excluding its obvious and critical importance in disturbing genome integrity and precipitating cancer) remains incompletely understood. Specifically, if DNA damage repair is the key and limiting mechanism in longevity and biological aging, one would expect that gain‐of‐function DNA repair mutants would extend maximal lifespan and that human longitudinal studies would demonstrate associations between increased maximal lifespan and superior DNA repair. Such evidence, however, has been scant and difficult to come by so far, with one recent study showing an increase in lifespan following overexpression of antioxidating enzyme that excludes 8‐oxoguanine from nucleic acids (De Luca *et al*., 2013) and with no human longitudinal data published as yet to the best of our knowledge. It is therefore important to assess how resilient is the DNA repair system and whether and how it combines with biological aging to produce measurable phenotypes.

One model that can be used to assess the impact of exposure to DNA damage is the use of whole‐body gamma irradiation (WBI), which results in systemic DNA damage (Simpkin, 1999) and hematopoietic cell death in a dose‐dependent manner (Grahn & Hamilton, 1957; Anderson & Warner, 1976). Hematopoietic cells are amongst the most radiosensitive cells in mammals and lymphocytes are among the most sensitive hematopoietic lineage cells. In mice, hematopoietic cell death is detectable at WBI doses of 0.5 gray (Gy), and severe systemic depletion is induced in the 4 Gy range (Anderson & Warner, 1976), with lethal outcome occurring between 7 and 10 Gy depending on the strain (Grahn & Hamilton, 1957). Specifically, C57BL/6 (B6) mice are estimated to be about twice as radioresistant as humans, in whom the same effects occur at half the doses mentioned above (rev. in Nias, 1990).

Barring severe host infection and death, surviving hematopoietic cells are mobilized to rapidly divide and eventually repopulate lymphoid and other compartments to pre‐irradiation levels (rev. in Anderson & Warner, 1976; Nias, 1990). While the kinetics of immune repopulation have long been known (Takada *et al*., 1969), long‐term immune homeostasis, function, and protection following repopulation remain relatively uncharacterized, particularly in humans. Hiroshima and Nagasaki bomb survivors were reported to display some hallmarks of increased or early immune aging (Nakachi *et al*., 2004; Yamaoka *et al*., 2004; Hayashi *et al*., 2005; Kusunoki & Hayashi, 2008). However, it is unclear whether these hallmarks translate directly into increased risks from new or emerging infections, and whether vaccination efficacy against such infections is significantly reduced.

In order to test vaccination and immunity in old age, we employed early‐life sublethal WBI. We then followed the animals longitudinally into the old age to examine immune response to a single‐cycle live vaccine, RepliVAX West Nile (R‐WN) (Widman *et al*., 2009), and protective immunity following a subsequent challenge with a potentially lethal dose of live West Nile virus (WNV). In unmanipulated mice, R‐WN vaccination is sufficient to provide strong protection to both adult and old mice against lethal WNV challenge (Uhrlaub *et al*., 2011), providing a good baseline with which to measure the potential exacerbation of immune aging‐induced by DNA damage. We found that, perhaps contrary to the expectations predicted by the DNA damage aging theories, a single WBI exposure in youth did not decrease late‐life survival from WNV challenge following vaccination. Vaccine and WNV‐specific immunity, including T‐cell and antibody responses, were not substantially affected by WBI dose in youth. Therefore, a single dose of nonlethal WBI in youth, resulting in widespread DNA damage and repopulation stress in hematopoietic cells, leaves no significant trace of increased immune aging and decreased immune function and protection in a lethal vaccine challenge model. These results are encouraging from the standpoint of defined, accidental exposure to sublethal ionizing radiation of the immune system.

## Results

### Hypotheses and experimental design

**Figure 1:**
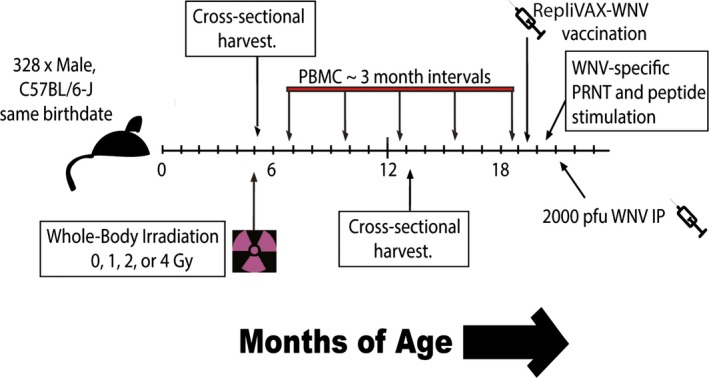
Experimental design of longitudinal cohorts. Age‐matched, adult, male, C57BL/six mice with identical birthdates were divided into groups receiving 0, 1, 2, or 4 gray (Gy) of WBI in a single dose. A cross‐sectional harvest of representative mice from each group was collected at 3 days following WBI. A similar cross‐sectional harvest occurred at 13 months of age, long after complete repopulation of immune cells. Immune populations in peripheral blood were tracked at 3‐month intervals from PBMC until 19 months of age. At 19 months of age, mice were injected with 10^5^ pfu RWN vaccine i.p. Immune function and antibody generation were assayed 45 days postvaccination. Sixty days postvaccination, mice were challenged with 2000 pfu WNV i.p. and mice were tracked for survival. This experimental design was completed in its entirety on two separate cohorts of mice, with birthdates separated by approximately 1 year.

If lifetime turnover and/or DNA damage‐related senescence are causative factors in hematopoietic immune aging, we hypothesized that profound genotoxic stress, imparted by WBI exposure, would increase immune aging phenotypes in a dose‐dependent manner. To test these hypotheses, we employed the experimental strategy outlined in Fig. 1. Age‐matched, adult, male B6 mice were divided into groups receiving 0, 1, 2, or 4 gray (Gy) of WBI in a single dose. (Another group of animals irradiated at 0.5 Gy was initially used in our first set of experiments, but that dose subsequently abandoned because it showed no substantial difference from 1 Gy group.) Mice were allowed to rest for 72 h, and six randomized mice from each group were euthanized to confirm systemic hematopoietic cell death in a WBI dose‐dependent manner across primary and secondary organs. No mice died as a result of WBI exposure at these doses, as would be expected from the WBI LD_50/30_ of this strain (Grahn & Hamilton, 1957). Another cross‐sectional harvest of representative mice from each group was collected at ~13 months of age, long after complete repopulation of immune cells. In addition, immune populations in peripheral blood were tracked at 3‐month intervals from WBI until 19 months of age. At 19 months of age, mice were injected with RWN vaccine, which we have previously shown to protect old mice from WNV (Uhrlaub *et al*., 2011). We examined effector responses to the vaccine, allowed for immune memory to form and mature for 60 days, and then challenged the mice with a potentially lethal dose of live WNV. During vaccination and challenge, WNV‐specific T‐cell and antibody immunity were assayed. This design was completed on two independent large cohorts of mice (Fig. 1).

### Sublethal WBI exposure in youth and T‐cell homeostasis in the old age

**Figure 2:**
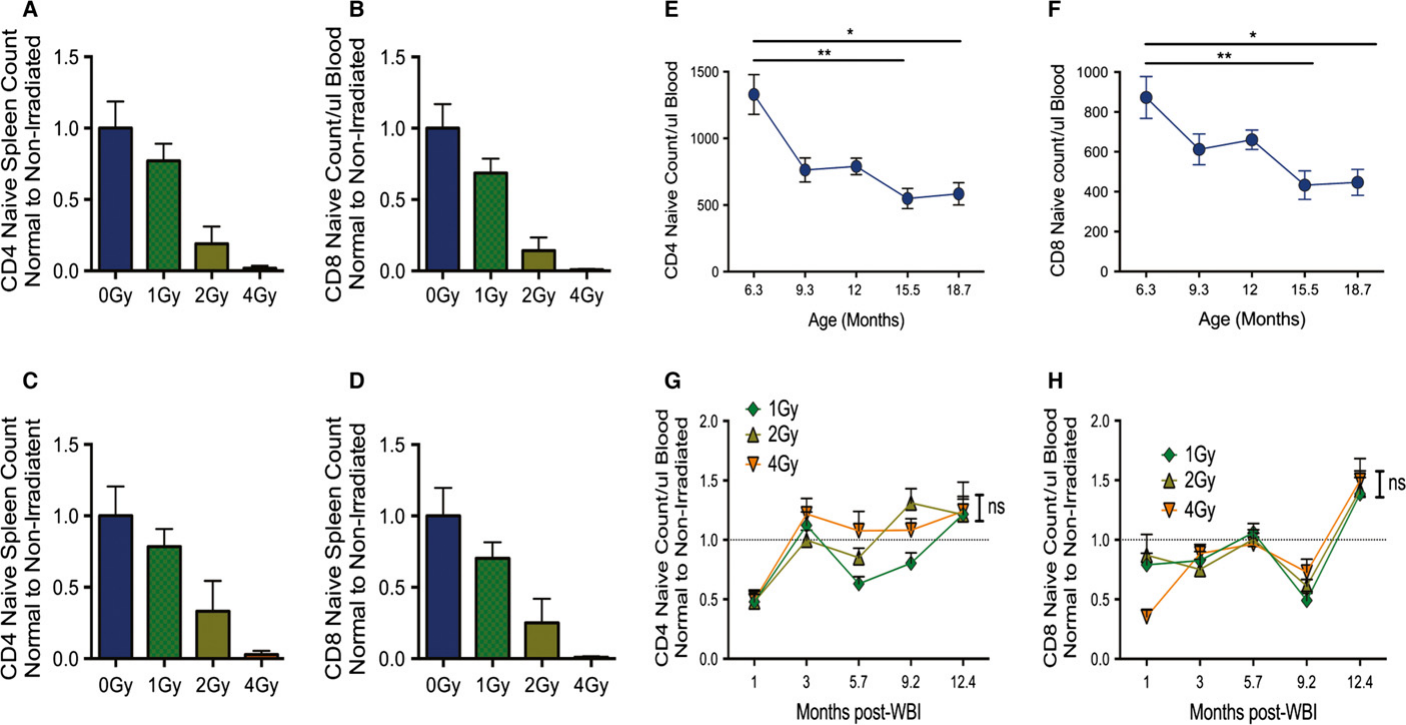
Sublethal WBI exposure in youth does not result in significantly depressed T‐cell counts in old age. Graphs shown from a single representative cohort. A–D: At 3 days postradiation, 4–6 mice in each radiation group were euthanized and analyzed for immune cell subset abundance in spleen and PBMC by flow cytometry in concert with cell counts. Shown are cell counts normalized to the average of counts of the 0 Gy (mock‐irradiated) control group. (A) Cell counts of Naïve CD4 T cells in spleen. (B) Cell counts for Naïve CD8 T cells in spleen. (C) Cell counts of Naïve CD4 T cells in peripheral blood. (D) Cell counts of Naïve CD8 T cells in peripheral blood. E–H: Immune populations in PBMC were harvested at ~3‐month intervals following WBI or mock irradiation, until mice were ~19 months of age. Ages in E, F correspond to time points in G, H, respectively. Shown are Tukey multiple comparison tests of two‐way anova between time points (E–F), or between radiation groups at the final time point (G–H). (E) Raw counts of Naïve CD4 T cells in peripheral blood over lifespan in unirradiated mice. (F) Raw counts of Naïve CD8 T cells in peripheral blood over lifespan in unirradiated mice. (G) Counts of Naïve CD4 T cells in peripheral blood over lifespan, normalized to the average of 0 Gy mice at each time point. (H) As in G, for Naïve CD8 T cells. Error bars represent SEM throughout.

Three days following WBI, spleen and blood were harvested from a subset of mice in each WBI dosage group. As expected, WBI resulted in cell death and depletion of a variety of immune cell subsets in a dose‐dependent manner across lymphoid tissues (Fig. 2A–D). We recently demonstrated that these irradiation doses cause the expected DNA damage, including histone marking by ATM kinase, in all assessed T‐cell and other nucleated blood cell subsets (Pugh *et al*., 2014). While we did not directly assess the damage to hematopoietic cells, our irradiation dose was carefully controlled by calibration and dose absorbance (Pugh *et al*., 2014; Seed *et al*., 2016) and was in the range documented by others to cause hematopoietic stem cell damage (Mohrin *et al*., 2010).

**Figure 3:**
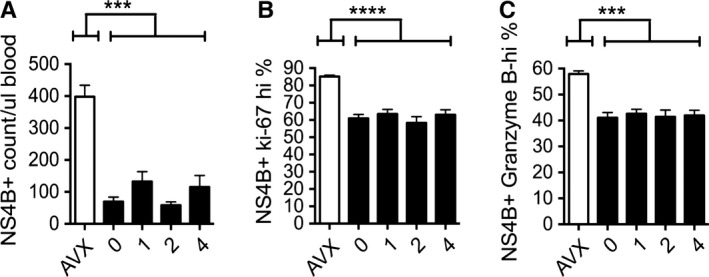
Acute vaccine response is equivalent in old mice exposed to WBI in youth. Graphs shown from a single representative cohort, *n* ≥ 8 per group. All mice injected with 10^5^ pfu RWN IP on the same day. AVX = adult vaccinated controls (5 months). All other groups ≥ 19 months. Numbers on *x*‐axis indicate dose of WBI in youth in Gy. All data from PBMC. All one‐way anova ≥ *. Results of Tukey multiple comparison test shown. (A) Counts of NS4B tet+ CD8 T cells on day 7 post‐RWN. (B) Percent of Ki‐67 hi, NS4B tet+ CD8 T cells on day 7 post‐RWN. (C) Percent of NS4B tet+ CD8 T cells that are Granzyme B‐hi on day 7 post‐RWN. Error bars represent SEM throughout.

Immune cell populations from PBMC were assayed throughout lifetime, starting 30 days following WBI. At 30 days post‐WBI, populations of CD4 and CD8 T_N_ cell counts remained depressed relative to nonirradiated controls (Fig. 2G,H). Similarly, B‐cell counts in PBMC had not fully recovered by 30 days (Fig. S2C). Radioresistant NK cells, however, had fully repopulated by 30 days following WBI (Fig. S2D). Typical declines in CD4 and CD8 T_N_ cells were observed with aging in mock‐irradiated mice (Figs. 2A and 3B). These declines were accompanied by slight increases in the total counts of central memory CD8 T cells (CM), and effector memory CD8 T cells (EM) over lifespan (Fig. S2G,H). However, counts of CD8 and CD4 T_N_ cells from irradiated mice were not depressed relative to mock‐irradiated controls by 19 months of age (Fig. 2G,H). There also were no significant differences by 19 months of age in peripheral counts of CD8 CM, CD8 EM, nor γδT cells among the groups, regardless of irradiation in youth (Fig. S2F–J). Counts of B cells in PBMC were depressed among mice that received 4 Gy in youth, relative to all other groups, by 19 months of age. However, B‐cell counts for that group also remained within the range of natural variation over lifespan (4 Gy mean at final time point = 5249 cells, 0 Gy range at initial time point 2410‐7406 cells) (Fig. S2D). Of importance, the extent of DNA damage, as judged by the histone marking (γH2‐AX), was unremarkable and did not differ with the dose of radiation exposure in youth in any of the T‐cell subsets examined (naïve, central, and effector memory, Fig. S3).

We conclude that irradiation doses up to 4 Gy do not significantly disturb long‐term homeostasis of the main lymphoid cell subsets in male B6 mice following repopulation.

### High‐dose sublethal WBI exposure in youth and effector and memory immune response to a live attenuated vaccine in the old age

NS4b_2488_ is the dominant CD8 T‐cell epitope of the WNV in B6 mice (Brien *et al*., 2007) and that epitope is conserved in the R‐WN vaccine (Widman *et al*., 2009; Uhrlaub *et al*., 2011), allowing us to follow the response to vaccination using this peptide complexed to soluble H‐2D^b^ multimers (Uhrlaub *et al*., 2011). On d7 postvaccination, absolute numbers of NS4b tetramer‐positive (NS4b+) CD8 T cells in all old groups of mice were significantly reduced compared with adult mice, confirming our previous observations (Uhrlaub *et al*., 2011) that primary responses against R‐WN are drastically reduced in old mice (Fig. 3A). However, we found no difference between the groups of old mice based upon the presence or dose of irradiation in youth – NS4b+ cells were equivalently abundant in the blood of old mice receiving no irradiation compared with mice receiving 1–4 Gy WBI (Fig. 3A). Anti‐Ki‐67 antibody marks cells in the cell cycle (G1, S, G2, or M phase), but not those in interphase (G0). Ki‐67+ NS4B+ cells were relatively equally distributed across all groups at the height of vaccination response (Fig. 3B). NS4b+ cells also displayed similar content of the lytic granule molecule Granzyme B, important in cytotoxic function of CD8+ cells, across all groups (Fig. 3C). We therefore conclude that the presence and the dose of irradiation in youth did not affect *in vivo* accumulation (total numbers, Fig. 3A), proliferation (Ki‐67+ %, Fig. 3B), or differentiation (% Granzyme B+ cells, Fig. 3C) of effector CD8 T cells at the peak of the response to a live attenuated vaccine in the old age.

**Figure 4:**
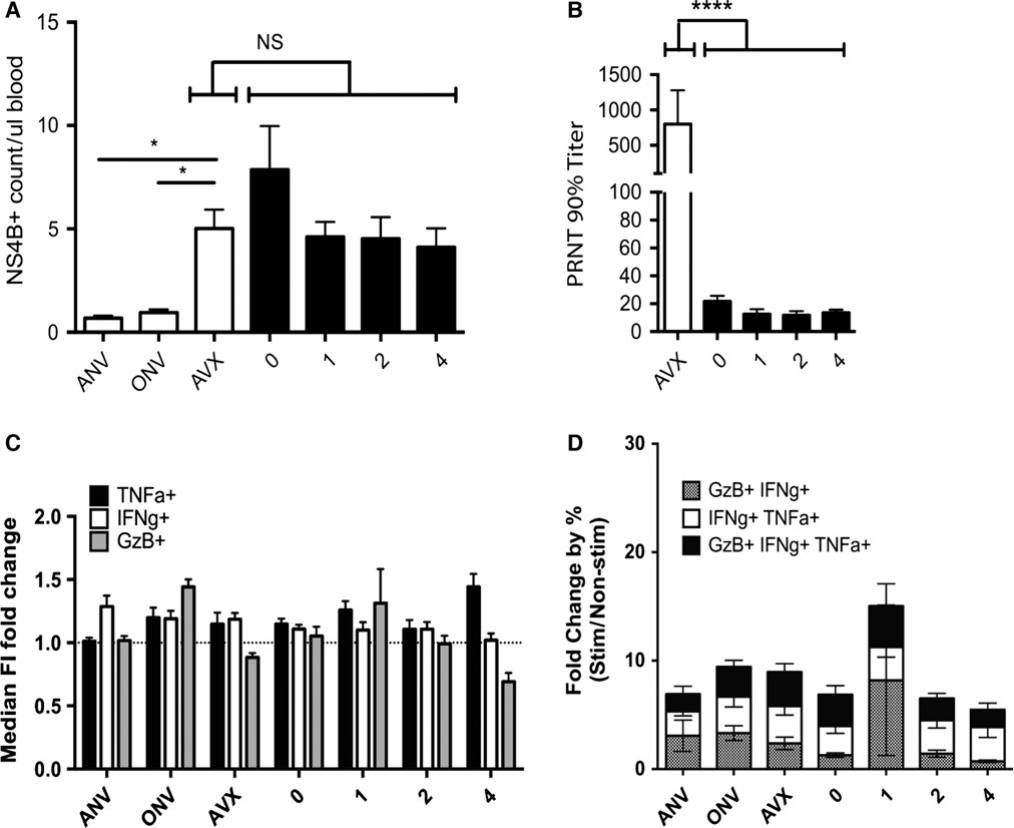
T‐cell function and vaccination efficacy are equivalent in old mice exposed to WBI in youth. AVX = adult vaccinated controls, ANV = adult nonvaccinated controls (5 months old). ONV = old nonvaccinated mice, age‐matched to cohort (21 months). Data shown from a single cohort. Numbers on *x*‐axis indicate dose of WBI in youth in Gy. *n* ≥ 10 per group, except ANV = 4. (A) Counts of NS4B tetramer‐positive cells from PBMC at day 45 post‐RWN vaccination. (B) Results of plaque reduction neutralization test of serum collected from mice 45 days post‐RWN vaccination. C–D: PBMC from mice on day 45 post‐RWN vaccination were isolated and stimulated with CD8‐specific WNV peptides for 5 h in the presence of Brefeldin A. (C) Fold change (peptides/no peptides) of individual cytokine production by median fluorescence intensity. (D) Fold change in the percentage of polyfunctional CD8 T cells (peptides/no peptides) of CD8 T cells, treated as in (C). Results of post‐tests of one‐way anova shown throughout. Error bars represent SEM throughout.

At 45 days postvaccination and prior to challenge, numbers of NS4b+ memory CD8+ T cells were evaluated and were found to not significantly differ between adult vaccinated controls and old vaccinated nonirradiated mice (Fig. 4A), further confirming our prior data that steady‐state memory set point is not different with age, despite drastic differences observed at the height of the acute effector response (Uhrlaub *et al*., 2011). Again, we found no difference in numbers of memory CD8+ NS4b+ cells between old control and 1–4 Gy irradiated mice at any irradiation dose used here (Fig. 4A). Similarly, functional humoral memory response against the virus assessed in the serum 45 days postvaccination showed no difference in old mice based on the dose of irradiation in youth (Fig. 4B). This was concluded based upon the analysis of the neutralizing potential of WNV‐specific antibodies using a plaque reducing neutralizing titer (PRNT) assay. While adult vaccinated mice showed on average 40‐fold higher PRNT titers compared with old nonirradiated counterparts, similar to our original observations (Uhrlaub *et al*., 2011), WBI dose in youth did not significantly alter the neutralizing potential of serum antibody generated by RWN vaccination in old mice (Fig. 4A). Finally, 45 days postvaccination, PBMCs were harvested and stimulated with WNV peptides recognized by CD8 T cells in the presence of Brefeldin A to evaluate the function of memory CD8+ cells (Fig. 4C,D). No significant differences were noted across WBI doses with regard to individual cytokine production (Fig. 4C), although Granzyme B levels exhibited a trend toward reduction at the highest irradiation dose (4 Gy). Similarly, percentages of cells producing multiple cytokines (polyfunctional cytokine production) mounted against WNV‐specific peptides were not different between the groups (Fig. 4D).

### Impact of high‐dose sublethal WBI exposure in youth upon WNV‐specific T‐cell responses following WNV challenge in vaccinated old mice

**Figure 5:**
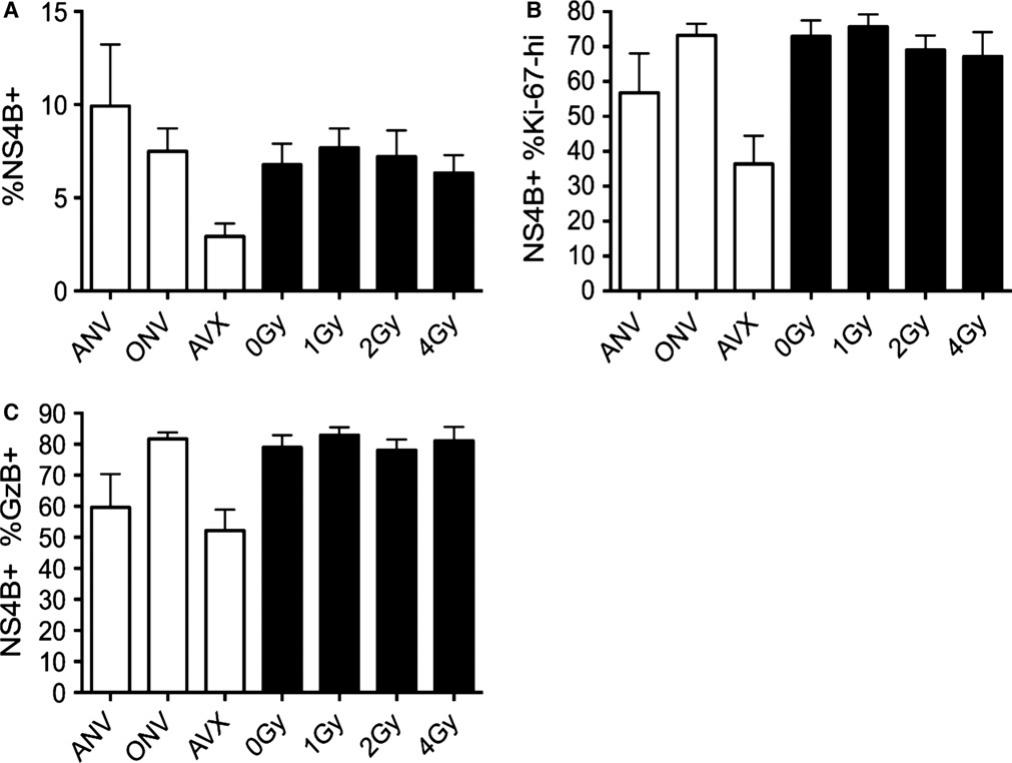
Responses to WNV challenge are equivalent in old mice exposed to WBI in youth. AVX = adult vaccinated controls, ANV = adult nonvaccinated controls (5 months old). ONV = nonvaccinated mice, age‐matched to cohort (21 months). Numbers on *x*‐axis indicate dose of WBI in youth in Gy. (A) Percent of NS4B tet+ CD8 T cells in PBMC on day 8 post‐WNV infection. (B) Percent of NS4B tet+ T cells that are Ki‐67‐hi on day 8 post‐WNV infection. (C) Percent of NS4B tet+ T cells that are Granzyme B‐hi on day 8 post‐WNV infection. Error bars represent SEM throughout.

Sixty days post‐RWN vaccination, mice were injected IP with a dose of WNV (2000 pfu) lethal to 100% of unvaccinated mice. At the height of WNV challenge, 8 days post‐WNV infection, NS4b+ populations expanded and responded with roughly equivalent size (Fig. 5A). Vaccinated adult controls exhibited a dampened T‐cell response compared with other groups because of superior antibody neutralization, as previously reported (Uhrlaub *et al*., 2011). Similarly, NS4b+ T‐cell proliferation was equivalent between all irradiation groups by proliferation marker Ki‐67 (Fig. 5B). Granzyme B production due to WNV challenge was also undisturbed by WBI dose in youth (Fig. 5C). Overall, we noted no significant lasting effects of WBI exposure in youth on WNV‐specific T‐cell abundance, proliferation, and function following WNV challenge in old age.

### Does high‐dose WBI in youth increase the risk of failed vaccination‐related death in the old age?

The ultimate measure of immune competence is the ability to resist infection. Therefore, following vaccination and lethal WNV challenge, mice were tracked for survival. That was particularly important because other players of the immune system whose numbers, but not function, were examined (CD4 T cells, NK cells, and TCRγδ cells), can play a role in WNV resistance in mice (rev. in Suthar *et al*., 2013).

**Figure 6:**
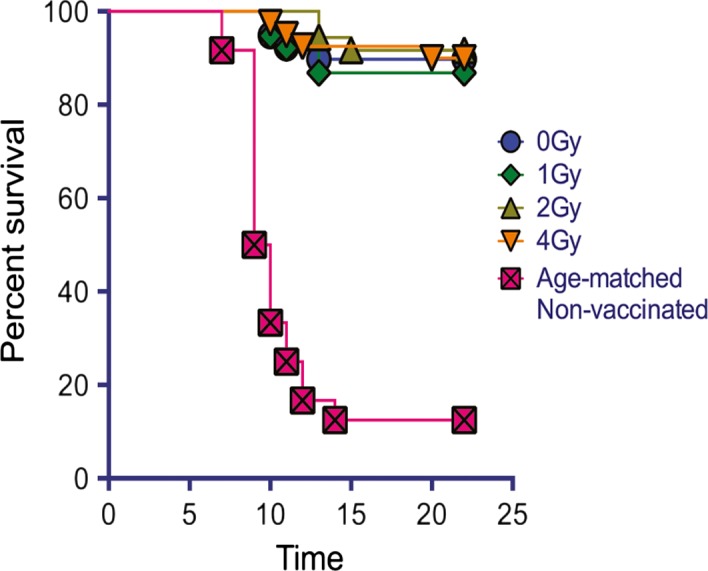
Survival from WNV challenge is equivalent in old mice exposed to WBI in youth. Survival shown following RWN vaccination at approximately 19 months of age, and WNV challenge at approximately 21 months of age. Mice were infected with 2000 pfu WNV IP. Graph is a combination of both cohorts (no group statistically different between cohorts). Shown are results of individual comparisons from Kaplan–Meier tests. *n* ≥ 12 per group. Kaplan–Meier between age‐matched nonvaccinated mice vs. all other groups: Difference between non‐vaccinated age matched mice and all other groups *p*<0.0001.

In concert with our other data, survival of old mice that received 1, 2, or 4 Gy of WBI in youth was not significantly different than mice that were mock‐irradiated in youth, nor was there a different trend in survival due to radiation dose (Fig. 6). We therefore conclude that exposure to up to 4 Gy of sublethal radiation in youth did not prevent or impair the adaptive antiviral immune response, from protecting against lethal WNV challenge following vaccination with a live attenuated vaccine.

## Discussion

In this report, we have systematically and longitudinally studied the impact in mice of early‐life exposure to sublethal WBI upon immune repopulation, homeostasis, response to vaccination, and ability to resist lethal challenge in the old age. The obtained results were exceedingly consistent and clear, and we conclude that a single, widespread DNA damage and repopulation stress in haematopoietic cells does not lead to increased aging immune phenotypes in mice. Because WBI applies DNA damage to both immune and nonimmune cells, our experimental design theoretically addresses both direct and systemic relationships between DNA damage and immune aging. Although increased unrepaired DNA damage correlates with aging in some hematopoietic populations (Nijnik *et al*., 2007), and DNA damage can cause senescence and inflammation in a variety of cell types (Rodier *et al*., 2009; Chandler & Peters, 2013), no immune aging effect was replicated by WBI alone in the present studies. There are several possible not mutually exclusive explanations for this observation. These are: (i) that the sum total of DNA damage is not a cause of immune aging, (ii) that the youthful capacity of DNA repair and apoptotic mechanisms are able to repair and nullify even severe DNA damage in early life, or (iii) that the response to gradual DNA damage that occurs over lifespan is fundamentally different than the response to DNA damage delivered by a single dose of WBI, and that only the former is of importance during aging. Experimental analysis with repeated, low‐level exposure to DNA damage will be necessary to test and distinguish between these hypotheses.

Some recent studies have used WBI as a model for aging in mice (Shao *et al*., 2014; Chang *et al*., 2016). These studies document similarities in HSC senescence between natural aging and WBI in youth, among other nonimmune phenotypes. Unfortunately, neither group tested immune function nor challenged reconstituted immune systems *in vivo*. Both studies employ higher doses of WBI than in our present study, in conjunction with HSC transfer models, reconstituting irradiated HSCs in lethally irradiated hosts. While this model is necessary for the focused study of HSCs, it unfortunately adds many confounding covariables to a direct comparison between natural immune aging and WBI exposure. For instance, we observed strong peripheral immune cell division and reconstitution in our model, even in Naïve T cells (Fig. S4), which would not be present in an HSC transfer model. Indeed, this factor may have led to irradiated populations having slightly higher counts of Naïve T cells in old age (Fig. 2). Taken together with our results, this suggests that WBI, although able to replicate senescence in HSCs and some nonimmune cell types, fails to additively impact other systemic immune aging phenotypes. A combination of HSC reconstitution and *in vivo* challenge models will be necessary in future studies to resolve some of the remaining issues.

Older individuals exhibit dampened primary effector responses to vaccination (rev. in. Nikolich‐Zugich, 2014). This age‐related defect in generating effector immunity was recapitulated in our experiments following R‐WN vaccination. However, the response to vaccine by old naïve NS4b+ cells was not further degraded by WBI up to 4 Gy. We did not note any increase in standing DNA damage in peripheral lymphocytes following repopulation (Fig. S3A), implying that either the surviving precursor cells with DNA damage were not contributing to repopulation, or that DNA damage was adequately neutralized through division/differentiation and apoptosis. We independently examined the increase in standing DNA damage by γH2AX in peripheral immune cells with age. No CD8 T‐cell subset exhibited increased standing dsDNA breaks with age (Fig. S3B), implying that standing DNA damage is not a major intrinsic factor in T‐cell aging defects, at least not within the limits of our experimental design and detection sensitivity. Taken together, this implies that DNA repair mechanisms in hematopoietic cells, combined with culling of damaged cells through apoptosis, are adequate to overcome both lifetime DNA damage and proliferation stress and a single, whole‐body extensive DNA damage event in specific pathogen‐free mice.

While the numbers of peripheral B and NK cells in old mice decreased in groups exposed to the highest dose of WBI (4 Gy; Fig. S2D–E), these factors did not influence survival. The less numerous B cells in the 4 Gy group still produced equally effective neutralizing antibody. NK cells are dispensable for survival from WNV (Shrestha *et al*., 2006), leaving open the possibility that WBI in youth may effect long‐term immune control of pathogens dominantly controlled by NK cells, such as pox and herpesviruses (Yokoyama, 2005; Vivier *et al*., 2011).

Regardless of the above deliberations, our results provide hope that most, if not all, subjects that survive WBI exposures similar to those found in accidental or atomic bomb incidents in youth, may be immunologically fit to a previously underappreciated levels deep into old age. Indeed, similar results were obtained in a recent study examining responses to influenza vaccine in survivors of the Hiroshima nuclear explosion, where seroprotection and seroconversion were not affected by the magnitude of radiation exposure in a cohort over 65 year of age (Hayashi, T. Heather E. Lynch, Susan Geyer, Kengo Yoshida, Keiko Furudoi, Keiko Sasaki, Yukari Morishita, Hiroko Nagamura, Mayumi Maki, Yiqun Hu, Ikue Hayashi, Seishi Kyoizumi, Yoichiro Kusunoki, Waka Ohishi, Saeko Fujiwara, Ivo Shterev, Janko Nikolich‐Zugich Donna Murasko, Gregory D. Sempowski, Kei Nakachi, in preparation). Overall, our results highlight remarkable resilience of the immune system to withstand extensive DNA damage and continue to competently operate for life.

## Experimental procedures

### Mice

Adult (< 6 month) male C57BL/six mice were acquired from Jackson Laboratories and held under specific pathogen‐free conditions in the animal facility at the University of Arizona (UA) for life. All experiments were conducted by guidelines set by the UA Institutional Animal Care and Use Committee. As needed, mice were euthanized by isofluorane and spleen was collected into complete RPMI supplemented with 5% or 10% fetal bovine serum (FBS). Blood was taken from the heart for cross‐sectional harvests. For longitudinal time points, blood was taken by retro‐orbital bleed from living, anesthesia‐free mice. Red blood cells were hypotonically lysed.

### Viruses and vaccine

West Nile virus (WNV): Strain 385‐99, a kind gift from Robert Tesh, was injected IP at 2000 pfu/mouse. RepliVAX WNV was injected IP at 10^5^ pfu/mouse. Verification of viral titer and production of RWN stock are described elsewhere (Uhrlaub *et al*., 2011).

### Peptide stimulation

Blood samples were taken at 45 days following RWN vaccination, hypotonically lysed, and stimulated *ex vivo* with a pool of: NS4b 2488‐2496, and E 347‐354, peptides (21st century Biochemicals, Marlborough MA, USA) both at 10^−6^ m. Stimulation took place over 6 h in the presence of BFA.

### Plaque reduction neutralization test (PRNT)

Serial dilutions of mouse serum (1:10 minimum) were incubated with 100 pfu/well live WNV from the same stock received by mice, in a 96‐well format, for 6 h at 4 °C. Samples were then applied to a monolayer of Vero cells also in 96‐well format and allowed to incubate at 37 °C with 5% CO_2_ for 25 h. Resulting monolayers were fixed with ice‐cold 50% acetone/50% methanol for 30 min at −20 °C and allowed to dry overnight. Resulting monolayers were assayed with anti‐WNV antibody clone EG16, a kind gift from Dr Michael Diamond (Washington University, St. Louis, MO) followed by peroxidase‐labeled goat anti‐mouse IgG (XPL Inc., Gaithersburg, MD, USA). Infectious lesions were visualized in a DAB reaction. The dilution factor necessary for 90% reduction in infectious lesions was established by hand count. The average of duplicate assays per mouse was used.

### Irradiation

Whole‐Body irradiation was performed on a Gammacell Cs^137^ source irradiator calibrated by in‐house physicist from the UA Health Sciences Center. Dosage was verified with thermal luminescence dosimeters (TLDs) (Landauer Inc., Glenwood, IL, USA) and TLDs from the Medical Radiation Research Center at the University of Wisconsin. Dosages fell within 5% of expected values. Effective dose rate ranged 70.2–68.36 cGy/min depending on the age of the source and distance. For whole‐body irradiation, a maximum of eight mice were placed in sterile RadDisks (Braintree Scientific, Braintree, MA, USA) with no separation. WBI occurred before noon on a light–dark cycle 7 am–7 pm.

### Flow cytometry

Prior to each collection, voltages were manually calibrated to a common template using Rainbow Beads (BD Biosciences, San Jose, CA, USA), to insure accurate MFI tracking over time. Fluorescent conjugated α‐Mouse antibodies against CD3(SK7), CD4(MCD0430), CD8a(S3‐6.7), CD62L(MEL‐14), CD44(IM7), α‐Ki‐67(B56), CD127(A7R34), KLRG1(2FI), CD86(GL‐1), B220(RM2630), NK1.1(PK136), CD49b(DX5), CD19(RM7717), IgM(II/41), and MHC‐ii(M5/114.15.2) were purchased from commercial sources. Tetramers against NS4b (H‐2D(b) – SSVWNATTA) were obtained from the National Institutes of Health Tetramer Core Facility. Staining occurred at 4C followed by fixation and permeabilization (FoxP3 kit; eBioscience, San Diego, CA, USA). Blood and spleen counts were performed using a Hemavet cell counter (Drew Scientific, Dallas, TX, USA). Samples were run on a Fortessa Flow Cytometer equipped with four lasers and using DiVa software (BD Biosciences). Compensation and analysis were performed using FlowJo software (Tree Star, Ashland, OR, USA).

### Statistics

Statistics were performed in Prism 6.0 (GraphPad Software, La Jolla, CA, USA). When data from multiple cohorts were combined for analysis, data from each cohort were normalized to the average of 0 Gy group from that cohort, to avoid cohort‐specific biases. Significance is noted as follows throughout: ns = not significant, ****P < 0.0001, ***P < 0.001, **P < 0.01, *P < 0.05. All error bars shown are SEM.

## Conflict of interest

The authors declare no conflicts of interest.

## Funding

Supported by the USPHS contract from the National Institute of Allergy and Infectious Diseases HHSN272200900059C, to the Radiation Effects Research Foundation (Nakachi, PI) and its subcontract to Dr. Nikolich‐Zugich.

## Author contributions

JLP designed and performed experiments and wrote manuscript; SAF, ASS, and JP‐T performed experiments; JP provided statistical analysis and advice; JLU performed experiments and designed assays; T.H. and K.N. provided critical advice; MJS designed experiments and edited manuscript; JN‐Z designed experiments, wrote, and edited manuscript.

## Supporting information


**Fig. S1** Gating strategies for flow cytometry.
**Fig. S2** Cell counts from peripheral blood over lifespan.
**Fig. S3** WBI does not result in lasting standing DNA damage in repopulated T cells, and aged T cells do not bear signs of increased standing DNA damage.
**Fig. S4** Peripheral turnover is a significant portion of repopulation following WBI in Naive CD8 T cells.
