# Supplementary material for: Acute systemic DNA damage in youth does not impair immune defense with aging
Source: Aging Cell. 2016 Apr 13;15(4):686–93. doi: 10.1111/acel.12478 (PMC4933672; doi:10.1111/acel.12478)
Supplement: Supplementary file 1 — Fig. S1 Gating strategies for flow cytometry. Fig. S2 Cell counts from peripheral blood over lifespan. Fig. S3 WBI does not result in lasting standing DNA damage in repopulated T cells, and aged T cells do not bear signs of increased standing DNA damage. Fig. S4 Peripheral turnover is a significant portion of repopulation following WBI in Naive CD8 T cells. [file ACEL-15-686-s001.pdf]

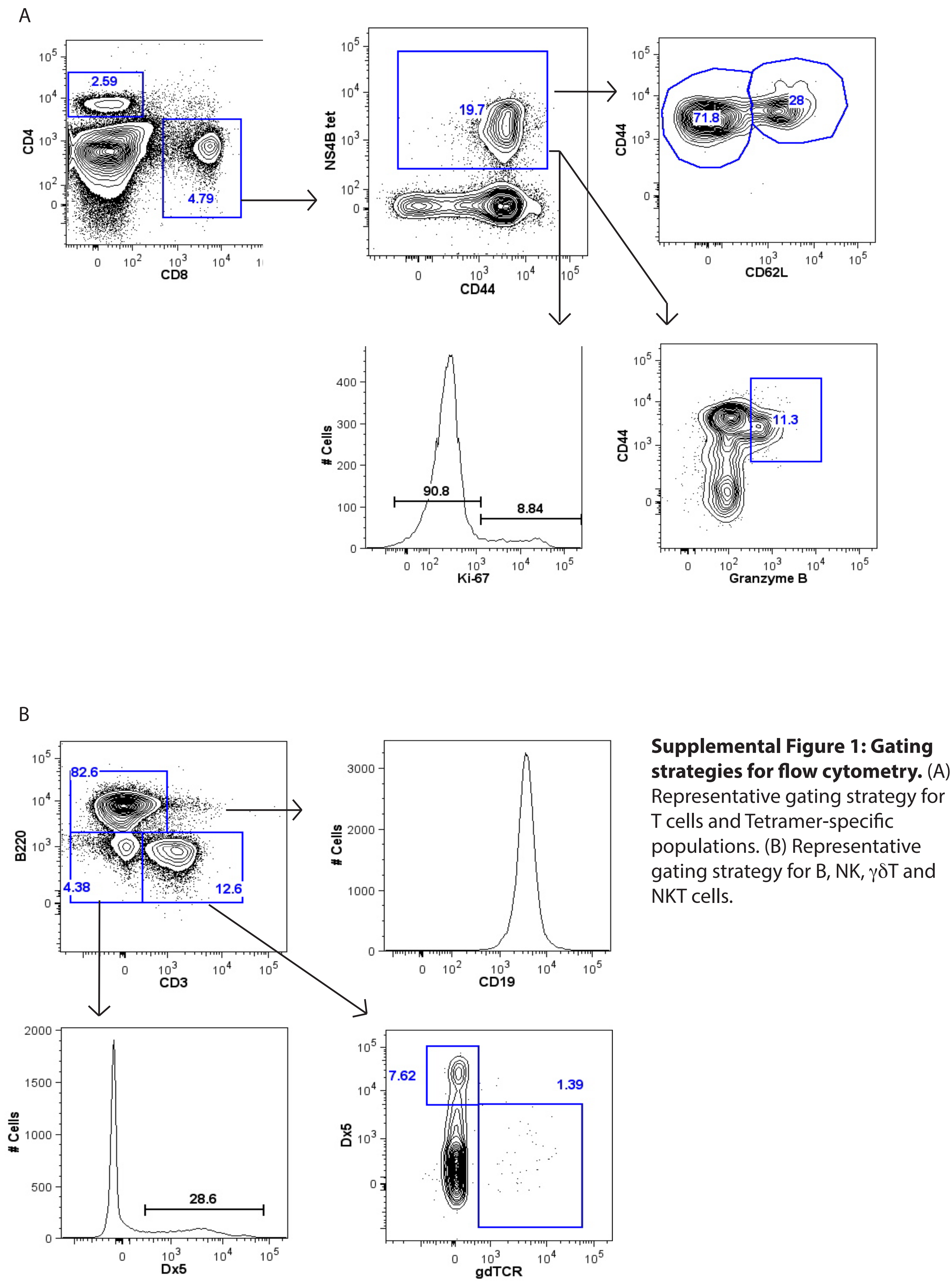

Supplemental Figure 1: Gating strategies for flow cytometry. (A) Representative gating strategy for T cells and Tetramer-specific populations. (B) Representative gating strategy for B, NK, γδT and NKT cells.

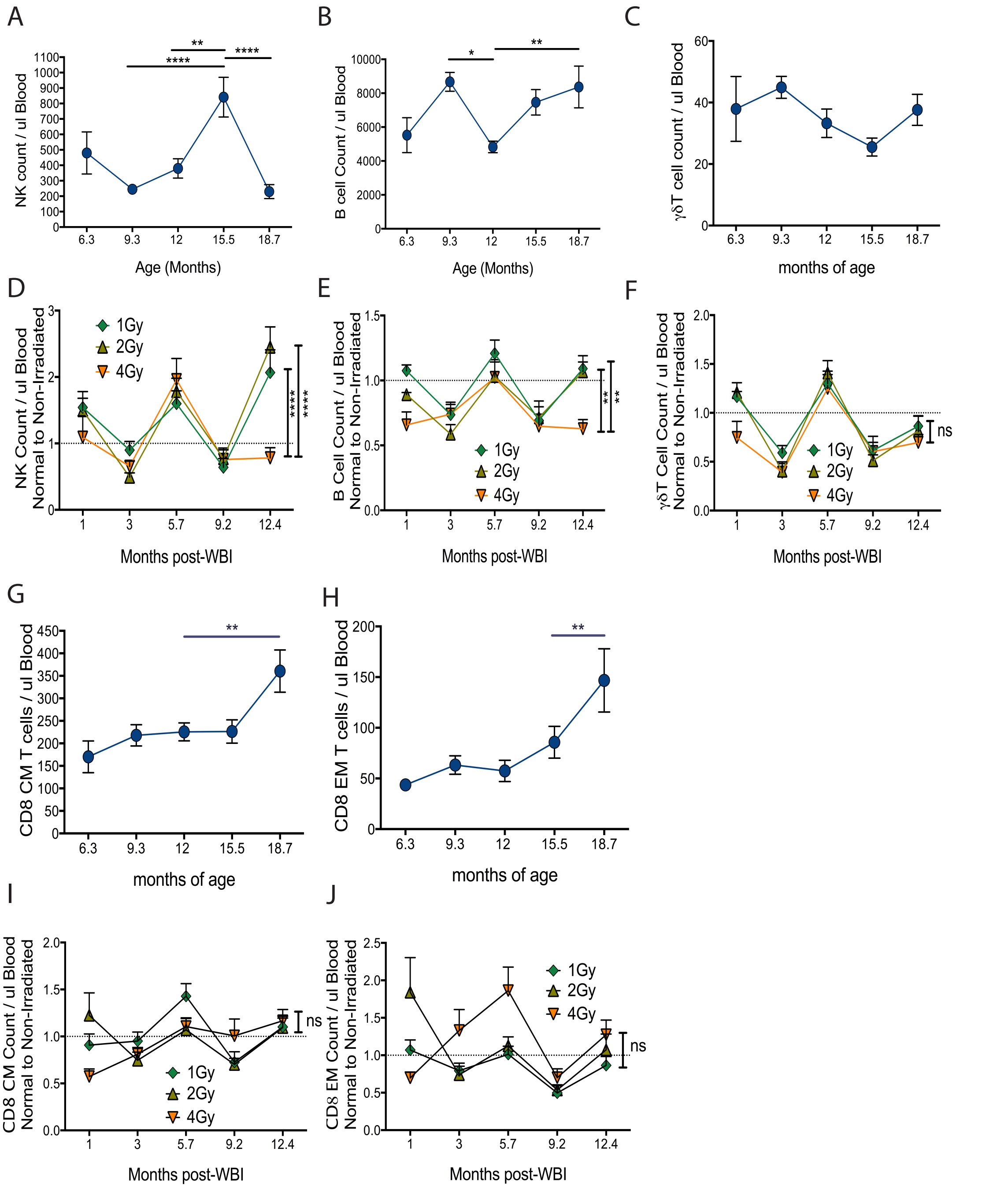

Supplemental Figure 2: Cell counts from peripheral blood over lifespan. Graphs shown from a single representative cohort. Immune populations in PBMC were harvested at ~3-month intervals following WBI at indicated doses, or mock-irradiation, until mice were ~19 months of age. For D,E,F,I,J, counts are normalized to mock-irradiated controls, shown directly above. Ages in A,B,C,G,H correspond to time points in D,E,F,I,J respectively. Shown are Tukey multiple comparison tests of 2-way ANOVA between time points (A,B,C,G,H) or between radiation groups at the final time point (G,E,F,I,J). (A) NK cell counts. (B) B cell counts. (C) γδT cell counts. (D) NK counts of irradiated mice, normalized to NK counts of mock irradaited mice. (E) as in D, for B cells. (F) as in D, for γδT cells. (G) as in A, for CD8 CM T cells. (H) as in A, for CD8 EM T cells. (I) as in D, for CD8 CM T cells. (J) as in D, for CD8 EM T cells.

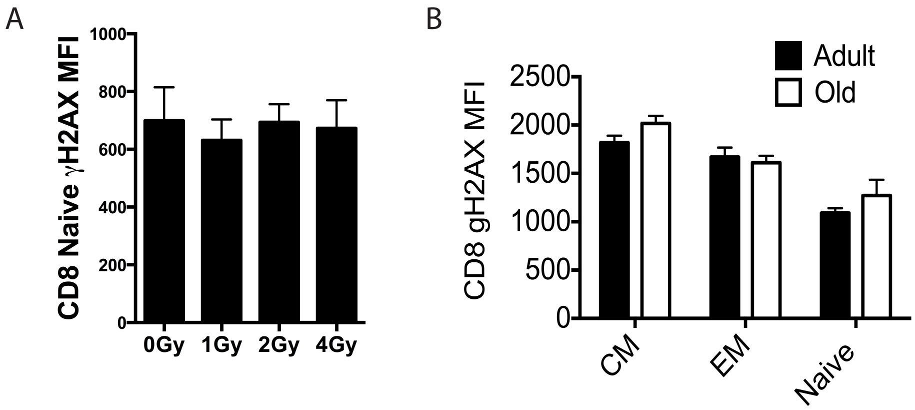

Supplemental Figure 3: WBI does not result in lasting standing DNA damage in repopulated T cells, and aged T cells do not bear signs of increased standing DNA damage. (A) Standing gH2AX MFI of Naive CD8 T cells from Spleens of mice at 13 months, approximately 8 months following WBI (second cross-sectional time point, as in Figure 1.) One-way ANOVA = ns. (B) Standing gH2AX MFI in old (18 months) and adult (5 months) mice for Central Memory, Effector Memory, and Naive CD8 T cells. Two -way ANOVA, Adult vs. Old = ns.

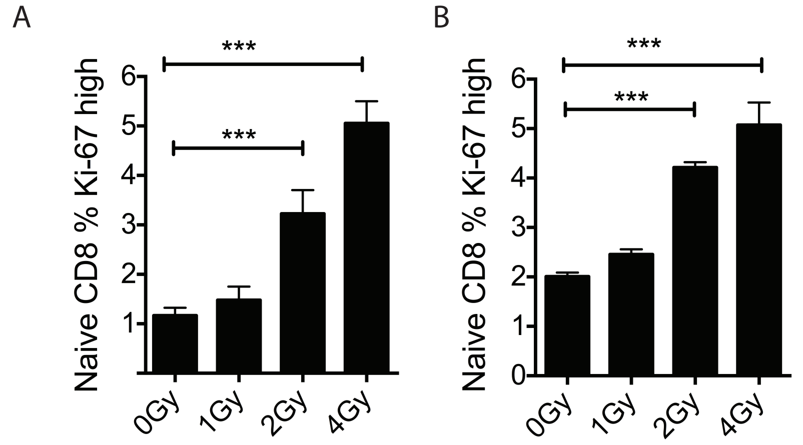

Supplemental Figure 4: Peripheral turnover is a significant portion of repopulation following WBI in Naive CD8 T cells. (A) Percent of Ki-67+ (dividing) Naive CD8 T cells from blood 3 days post-WBI at the indicated doses. n = 6 per group. (B) As in (A), 30 days post-WBI. N ≥ 12 per group. Shown are the results of Bonferroni post-tests between indicated groups. (Not all significance shown for clarity.) One-way ANOVA of both graphs ≤ ***.
